# Sedline^®^ Miscalculation of Depth of Anaesthesia Variables in Two Pigs Due to Electrocardiographic Signal Contamination

**DOI:** 10.3390/ani13172699

**Published:** 2023-08-23

**Authors:** Alessandro Mirra, Darren Hight, Alan Kovacevic, Olivier Louis Levionnois

**Affiliations:** 1 Section of Anaesthesiology and Pain Therapy, Department of Clinical Veterinary Medicine, Vetsuisse Faculty, University of Bern, 3012 Bern, Switzerland; olivier.levionnois@unibe.ch; 2 Department of Anaesthesiology and Pain Medicine, Inselspital Bern University Hospital, University of Bern, 3010 Bern, Switzerland; darren.hight@insel.ch; 3 Small Animal Internal Medicine, Vetsuisse Faculty, University of Bern, 3012 Bern, Switzerland; alan.kovacevic@unibe.ch

**Keywords:** Sedline, patient state index, suppression ratio, electroencephalogram, electrocardiogram, depth of anaesthesia, signal contamination, interference, pig

## Abstract

**Simple Summary:**

The assessment of the depth of anaesthesia remains challenging. In experimental pigs, the use of electroencephalography-based monitors has spread to characterizing brain activity depression as a surrogate of anaesthetics’ hypnotic effect. However, these devices have been validated for humans and, most of the time, a mere application has been performed in animal species including pigs with little effort made to assess their actual usability. Moreover, while artefacts within the electroencephalographic signal originating from other sources than the brain (e.g., electromyographic activity) have been extensively reported in humans, such interferences have not been characterized in this species yet. We report the contamination of the electroencephalographic signal by the electrocardiographic activity in two pigs undergoing general anaesthesia, leading to miscalculation of the variables displayed by the depth of the aesthesia monitor Sedline. Visual interpretation of the raw electroencephalogram remains necessary to avoid making wrong clinical decisions based on such electroencephalography-derived variables.

**Abstract:**

Two young (11-week-old) pigs underwent sole propofol anaesthesia as part of an experimental study. The depth of anaesthesia was evaluated both clinically and using the electroencephalography(EEG)-based monitor Sedline; in particular, the patient state index, suppression ratio, raw EEG traces, and its spectrogram were assessed. Physiological parameters and electrocardiographic activity were continuously monitored. In one pig (Case 1), during the administration of high doses of propofol, the Sedline-generated variables suddenly indicated an increased EEG activity while this was not confirmed by observation of either the raw EEG or its spectrogram. In the second pig (Case 2), a similar event was recorded during euthanasia with systemic pentobarbital. Both events happened while the EEG activity was isoelectric except for signal interferences and synchronous in rhythm and shape with the electrocardiographic activity. The suggestion of increased brain activity based on the interpretation of the Sedline variables was suspected wrong; most probably due to electrocardiographic interferences. In pigs, the patient state index and suppression ratio, as calculated by the Sedline monitor, could be influenced by the electrocardiographic activity contaminating the EEG trace, especially during otherwise isoelectric periods (strong EEG depression). Visual interpretation of the raw EEG and of the spectrogram remains necessary to identify such artefacts.

## 1. Introduction

The accurate assessment of the depth of anaesthesia (DoA) remains a significant challenge which is of major significance in pigs undergoing surgical experimental procedures. Indeed, DoA is still mainly monitored by assessing cardio-respiratory variables, trigeminal reflexes (e.g., palpebral reflex), and more complex responses (e.g., movement following a painful stimulus) [1,2,3]. To date, no gold standard methodology has been established. Over the last 20 years, some research groups attempted to improve DoA assessment in pigs by characterizing the extent of brain activity depression [4,5,6]. Different electroencephalographic(EEG)-based monitors have been used. However, being developed on human data and using proprietary algorithms, their inter-species translation could lead to signal miscalculation and misinterpretation. This methodology also presents limitations. For instance, non-GABAergic drugs (e.g., ketamine) have been shown to lead to erroneous increases in index values in humans [7,8] and little information is present concerning pigs. Furthermore, artefacts within the EEG originating from other sources than the brain (e.g., electrocardiographic (ECG) activity and eye movements) have been extensively reported in humans [9,10,11] and may differ in pigs. Despite this, few efforts have been made to characterize and remove interferences during application of these devices in animal species. The purpose of this case report in two pigs undergoing general anaesthesia is to describe interferences within the EEG, very likely due to the ECG signal, leading to miscalculation of the variables displayed by the Sedline monitor.

## 2. Case Presentation

### 2.1. Case 1

An 11-week-old, 27.1 kg, male castrated pig (phenotypic Edelschwein) underwent a sole propofol anaesthesia as part of an approved experimental study (protocol number from Canton Bern: BE116/19).

**Figure 1:**
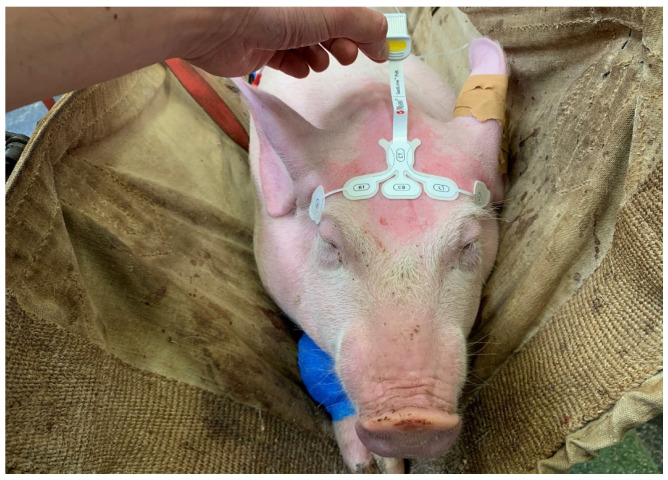
Positioning of the paediatric SedLine electrodes in a pig. The electrode line (L2, L1, R1, R2, from left to right) was placed over the frontal bone, keeping the rostral border of the electrodes on an imaginary line running between the lateral canthi of the eyes. The central GB (ground) and the caudal CT (reference) electrodes were placed on the mid-sagittal line.

**Figure 2:**
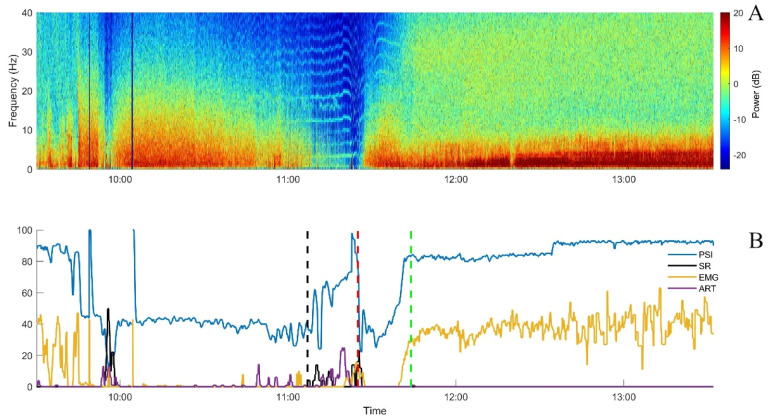
Spectrogram (**A**) and Sedline parameters graphically represented (**B**) from Case 1 over time. All Sedline values are smoothed using a 10 s median filter. Endotracheal intubation attempts occurred immediately prior to 10:00. Black dashed vertical line: sixth bolus of propofol; red dashed vertical line: propofol stop; green dashed vertical line: extubation; PSI: patient state index (blue, no unit); EMG: electromyographic activity (yellow, in %); SR: suppression ratio (black, in %); ART: artefact (purple, in %).

After acclimatization, the pig was placed in a sling and instrumented. Following application of a local anaesthetic cream (EMLA 5%, Anesderm, Pierre Fabre, Switzerland), an ear venous and arterial catheter were placed. A six lead wireless device (Televet 100, Engel Engineering Services GmbH, Heusenstamm, Germany) was used to continuously assess the ECG activity. A Sedline monitor (Masimo Corp., Irvine, CA, USA) was used to continuously assess the EEG activity and record the patient state index (PSI, no unit), suppression ratio (SR, in %), left and right spectral edge frequencies 95% (SEF L and SEF R, in Hz), as well as electromyographic activity (EMG, in %), and non-brain related artefacts (ARTF, in %) every two seconds. A peadiatric RD Sedline-sensor was placed over the frontal bone of the animal as previously reported [12] (Figure 1). The EEG data were exported (sampling rate: 178 Hz, left pre-frontal (Fp1) electrode) and spectrograms were created using two second moving windows (overlap 1-s; Figure 2).

The heart rate (HR), respiratory rate, arterial blood pressure, palpebral reflex, jaw tone, peripheral oxygen saturation (SpO_2_), and hind-limb nociceptive withdrawal reflex threshold (NWR; PainTracker; Dolosys GmbH, Berlin, Germany) were also assessed.

**Figure 3:**
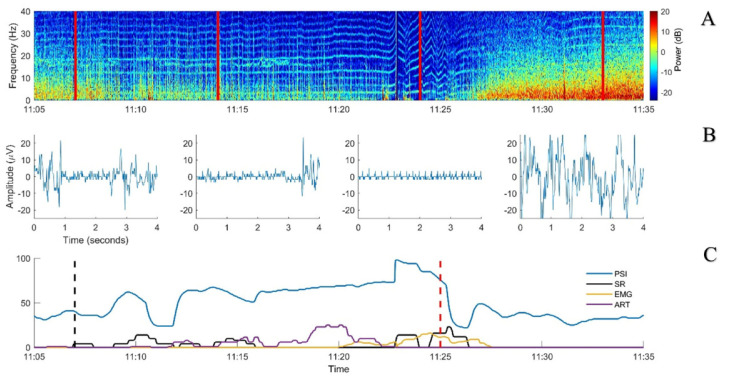
Period of suspected Sedline miscalculation from Case 1. (**A**) Spectrogram from the sixth propofol bolus (black dashed vertical line in 3C) until propofol was discontinued (red dashed vertical line in 3C). (**B**) Examples of 4-s EEG signals taken from the timepoints shown by the red full lines on the spectrogram. (**C**) Sedline parameters smoothed with a 10 s median filter. PSI: patient state index (blue, no unit); EMG: electromyographic activity (yellow, in %); SR: suppression ratio (black, in %); ART: artefact (purple, in %).

According to the experimental protocol, endotracheal intubation (ETI) was achieved with intravenous propofol to effect (approximately 5 mg/kg, administered at around 09:50). Simultaneously, a propofol infusion (20 mg/kg/h) was started and then incremented (6 mg/kg/h, associated with a bolus of 0.5 mg/kg, every 10 min) until reaching a target SR between 10 and 30%, as displayed on the Sedline. After each of the first five increments, a decrease in the PSI occurred, as expected. After the sixth one, the PSI increased markedly while the EEG appeared clearly suppressed on all four channels (soon after 11:00). No clinical signs of anaesthesia lightening were present: no gross movement, no palpebral reflex, absence of jaw tone, and stable cardiovascular and respiratory parameters. Moreover, the NWR threshold continued to increase. In parallel, the value for artefact recognition increased (max value 31%; Figure 3).

The heart rhythm was always sinusal, with a rate ranging between 180 and 200 beats per minute (bpm). The raw EEG displayed a nearly isoelectric line (<10 µV) in addition to monomorphic peaks at very regular intervals with a rate similar to the heart rhythm (Figure 3).

During the following 15 min, the SR showed some increases but did not reach the target to stop propofol administration (10–30%; Figure 3). However, DoA was considered clinically as deep and the EEG was judged as isoelectric. The displayed PSI and SR values were both considered aberrant, most probably due to the ECG signal appearing in the EEG. Therefore, propofol administration was stopped. A few minutes later, the PSI returned to values similar to those immediately before the sixth bolus of propofol and then increased progressively as the pig recovered from general anaesthesia (Figure 2). The recovery was uneventful and no other incongruences between clinical DoA assessment and Sedline-generated variables were noticed.

### 2.2. Case 2

An 11.5-week-old, 32.8 kg, female pig (phenotypic Edelschwein) underwent a similar experimental study as described above until recovery from general anaesthesia followed by final euthanasia while the pig was still instrumented. By the end of recovery, the pig started to show marked excitation and accidentally displaced its ear venous catheter. It was decided to proceed to euthanasia. As planned, pentobarbital (150 mg/kg) was administered but into the coccygeal arterial catheter which was still available. Injection was performed over 10 seconds (at 13:57). Immediately after drug administration, EEG and invasive blood pressure curves became isoelectric, the animal stopped breathing, and neither movements nor reflexes were present. However, the ECG activity continued to be present, showing different subsequent patterns:

- 13:59: third degree atrio-ventricular block (HR 120 bpm);
- 14:02: narrow QRS rescue rhythm (HR 96–108 bpm);
- 14:05: wide QRS rescue rhythm (HR 96–108 bpm) including a change in polarity associated with non-conducted P waves;
- 14:08: further change in polarity;
- 14:10–14:22: short periods of ventricular standstill and QRS waves with different polarity while atrial activity was still present.

**Figure 4:**
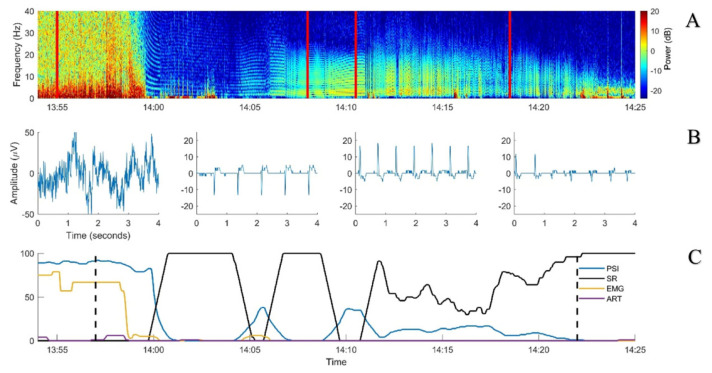
Spectrogram: example EEG sections and Sedline parameters over time from Case 2. (**A**) spectrogram of the EEG collected during euthanasia with intravenous pentobarbital (13:57). (**B**) Examples of 4-s EEG signals taken from the left pre-frontal electrode (Fp1) at four different timepoints corresponding to the vertical red bold lines on the spectrogram. (**C**) Sedline parameters smoothed with a 10 s median filter. First black dashed vertical line: first pentobarbital bolus for euthanasia. Second black vertical dashed line: intracardiac pentobarbital bolus. PSI: Patient State Index (blue, no unit); SR: suppression ratio (black, in %); EMG: electromyographic activity (yellow, in %); ART: artifact (purple, in %).

In the meantime, the ECG patterns were recognizable on the raw EEG traces and the PSI and SR values increased and decreased over time (Figure 4).

Due to the persistent ECG activity, 25 minutes after the induction of euthanasia (14:22), pentobarbital (150 mg/kg) was repeated intracardially. The ECG electrical activity ceased immediately, no further interferences on the EEG trace were noticed (flat isoelectric line), PSI decreased to 0, and SR increased to 100%.

## 3. Discussion and Conclusions

In this case report, we have shown that electrical activity generated from another source than the brain can lead to PSI and SR miscalculation by the Sedline monitor in pigs. The ECG signal contaminating the EEG was interpreted by the Sedline as an increased brain activity raising and decreasing the PSI and SR values, respectively. This miscalculation could lead to inadequate interpretation and clinical decision from the anaesthetist if the mere EEG-derived parameters are used without interpreting the raw EEG.

Many signals have been reported to interfere with the EEG, including electromyographic [9] and epileptiform activity [13], cerebral spinal fluid pulsation [14], and ECGs [15]. Of these, the latter has been the focus of many studies trying to reduce its interference with the EEG signal [16,17,18]. However, no gold standard methodology has been found yet. Moreover, even though most commercial EEG monitors claim to be able to reduce interferences from external noises, their algorithms remain proprietary; thus, it is difficult to judge their efficacy while attempting to estimate hypnotic depth in pigs. It is also important to mention that these devices have all been developed on and for humans and have never been validated in pigs [19]. Thus, their use in non-human species can result in inappropriate output and wrong clinical decisions.

The species-specific anatomical differences, as for example the proximity between brain and heart and the length of the neck, may enhance the risk of ECG signal contamination in the EEG dataset. This topic, as well as appropriate solutions, requires further investigations.

A previous study reported external signal interference involving artery pulsation while using the Sedline monitor [10]. In our animals, the interference appeared to be related to the ECG activity. In the first case, an increase in the PSI was noticed while propofol was administered at high dose. This increase would be expected to correlate with a lighter anaesthetic level but this did not mirror the dosage administered and the other clinical and neurophysiological (NWR threshold) parameters that reflected a deep anaesthetic plane. Visual inspection of the raw EEG signal revealed ECG interferences also present at many other time points from the beginning of the recording on. However, they seemed not to influence the Sedline calculations, probably due to their lower relative amplitude compared to the EEG signal. When brain activity became suppressed, the ECG became dominant in the EEG trace and was interpreted by the Sedline as an increase in its activity (Figure 3). A similar situation was found in the second case where the ECG was the sole electrical signal present after euthanasia. Also in this case, some ECG artefacts could be noticed in the spectrogram but they seemed not to influence the Sedline calculation until euthanasia was performed.

It is worth noting that the light blue or green horizontal stripes in the spectrogram, clearly visible when the EEG power is low, are most probably artefacts resulting from the inability of the Fast Fourier Transform (FFT) to model the low-frequency, sharp, and high-amplitude waves that the ECG signal produces [20].

In pigs, Sedline PSI and SR could be influenced by both the amplitude and polarity of the ECG trace contaminating the EEG, especially during isoelectric periods. Visual interpretation of the raw EEG and of the spectrogram remains necessary to identify such artefact interactions.

## Data Availability

The data presented in this study are openly available in Boris Portal at https://doi.org/10.48620/114.
